# A Thin Film Nanocomposite Membrane with MCM-41 Silica Nanoparticles for Brackish Water Purification

**DOI:** 10.3390/membranes6040050

**Published:** 2016-12-06

**Authors:** Mohammed Kadhom, Jun Yin, Baolin Deng

**Affiliations:** 1 Department of Chemical Engineering, University of Missouri, Columbia, MO 65211, USA; makbq6@mail.missouri.edu; 2 Department of Civil and Environmental Engineering, University of Missouri, Columbia, MO 65211, USA; jy3d9@mail.missouri.edu

**Keywords:** reverse osmosis (RO), thin film nanocomposite (TFN) membrane, MCM-41 nanoparticles (NPs)

## Abstract

Thin film nanocomposite (TFN) membranes containing MCM-41 silica nanoparticles (NPs) were synthesized by the interfacial polymerization (IP) process. An *m*-phenylenediamine (MPD) aqueous solution and an organic phase with trimesoyl chloride (TMC) dissolved in isooctane were used in the IP reaction, occurring on a nanoporous polysulfone (PSU) support layer. Isooctane was introduced as the organic solvent for TMC in this work due to its intermediate boiling point. MCM-41 silica NPs were loaded in MPD and TMC solutions in separate experiments, in a concentration range from 0 to 0.04 wt %, and the membrane performance was assessed and compared based on salt rejection and water flux. The prepared membranes were characterized via scanning electron microscopy (SEM), transmission electron microscopy (TEM), contact angle measurement, and attenuated total reflection Fourier transform infrared (ATR FT-IR) analysis. The results show that adding MCM-41 silica NPs into an MPD solution yields slightly improved and more stable results than adding them to a TMC solution. With 0.02% MCM-41 silica NPs in the MPD solution, the water flux was increased from 44.0 to 64.1 L/m^2^·h, while the rejection virtually remained the same at 95% (2000 ppm NaCl saline solution, 25 °C, 2068 kPa (300 psi)).

## 1. Introduction

Because of the rapid increase in human population in the last decades, the demand for fresh water has dramatically increased [1]. Desalination of sea and brackish water represents a major approach to supply fresh water for drinking and for agricultural and industrial applications [2]. Many large-scale desalination plants have been built around the world in recent years, and this trend is anticipated to continue [1,3]. Reverse osmosis (RO) is the most frequently selected desalination process for new desalination capacity buildup because of its reliability, inexpensiveness, and greenness [4]. Many studies have been conducted to improve the RO process and reduce energy consumption [1,4].

A thin film composite (TFC) membrane has been commonly used for desalination since it was developed 35 years ago [5]. It is mostly made via the interfacial polymerization of *m*-phenylenediamine (MPD) and trimesoyl chloride (TMC) on a polysulfone (PSU) or polyethersulfone (PES) support layer [5,6]. TFCs with other support such as modified polyvinylidene fluoride (PVDF) have also been developed [7].

Recently, an active effort has been devoted to develop thin film nanocomposite (TFN) membranes in which nano-sized additives are incorporated. Some nano additives have been found to improve membranes properties [8], particularly, hydrophilic nanomaterials [9,10]. For instance, hydrophilic zeolite particles have been shown to improve water permeation flux and decrease the contact angle. Zeolite nanoparticles (NPs) are assumed to have narrow pores that can allow the water to pass through and reject salt ions [11,12,13,14,15,16]; however, the magnitude of water flux capable of passing through very small micropores (<1 nm) needs to be further examined. Titanium dioxide (TiO_2_) was shown to enhance water flux as well when it was used in membrane manufacturing; in particular, when it is exposed to UV light, TiO_2_ becomes a superhydrophilic material [17]. Many researchers have added TiO_2_ NPs to TFNs in different forms [18,19,20,21]. Moreover, metal-organic frameworks (MOFs) have been used as fillers in different membrane applications, specifically for gas separation and purification [22].

Silica NPs have been injected as hydrophilic fillers in membranes and their support sheets, and have shown remarkable effects in both cases [8,23,24,25,26]. In a previous study by our group, two types of MCM-41 silica NPs were prepared—porous and non-porous—and it was demonstrated that porous MCM-41 silica NPs with internal pores of approximately 3 nm yielded better results than the non-porous one. By adding porous silica NPs, the water flux was significantly increased with almost no change in salt rejection. This is attributed to the enhancement of the membrane’s surface hydrophilicity and the short flow paths through the mesoporous structure [8].

In this work, porous MCM-41 silica NPs approximately 100 nm in diameter were added to either MPD or TMC solutions, and their impacts on the membrane performance were investigated. Different weight ratios of the fillers to the solutions were used. Additionally, for the first time, isooctane (2,2,4-Trimethylpentane), an organic solvent that has lower volatility than hexane and is safer to work with, was used to prepare the TMC solution. Contact angle, scanning electron microscopy, transmission electron microscopy, and attenuated total reflection Fourier transform infrared spectroscopy were used to characterize the membrane’s physicochemical properties.

## 2. Materials and Methods

### 2.1. Materials

Tetraethyl orthosilicate (TEOS, 98%, Sigma-Aldrich, St. Louis, MO, USA) and cetyltrimethylammonium bromide (CTAB, 95%, Aldrich) were used as silica raw material and surfactant, respectively, to prepare MCM-41 NPs. 2,2,4-Trimethylpentane (isooctane, 99%) was obtained from Fisher Scientific (Pittsburgh, PA, USA). Triethylamine (TEA, ≥99%) and (1s)-(+)-10-camphorsulfonic acid (CSA, 99%) used in CSA/TEA salt synthesis were purchased from Sigma-Aldrich. Sodium hydroxide (NaOH) and calcium chloride (CaCl_2_) were obtained from Fisher Scientific. The casting solution used to make the PSU support layer was prepared by dissolving polysulfone pellets (PSU, *M_W_* = 35,000, Sigma-Aldrich) in *N*,*N*-dimethylformamide (DMF, 99.8%, Sigma-Aldrich). *m*-Phenylenediamine (MPD, ≥99%, Fisher) and trimesoyl chloride (TMC, ≥98.5%, Sigma-Aldrich) were used in membrane preparation. The Millipore DI water (18.2 MΩ·cm, produced by Synergy 185, EMD Millipore Corp., Billerica, MA, USA) was used to prepare solutions and for rinsing and cleaning.

### 2.2. Preparation and Characterization of MCM-41 NPs

The MCM-41 NP preparation procedure has been reported in the literature [27]: 3.5 mL of a 2 M NaOH solution was added to 480 mL of DI water and mixed well for 10 min. Then, the solution was heated to 353 K, and 1.0 g of CTAB was added and mixed for 30 min. Next, 5 mL of TEOS was added drop by drop, and the mixture was left to stir. After 2 h, a white slurry was formed. The mixture was centrifuged (Eppendorf, Hamburg, Germany, centrifuge 5810 R) for 10 min at 10,000 rpm under a temperature of 20 °C, and the solid product was washed and centrifuged again twice. The product was collected, left to dry at room temperature, and then calcinated in a furnace for 4 h at 823 K.

### 2.3. Preparation of PSU Support Layer Sheets

The PSU sheets were manufactured using the phase inversion phenomenon. The casting solution was prepared by dissolving 15 wt % of PSU in a DMF solvent and heating to 60 °C under stirring for 5 h. A clear solution thus formed was kept overnight for degassing. The membrane sheets were fabricated by spreading the solution on a glass plate and casting by a casting knife (EQ-Se-KTQ-150, MTI Corp., Richmond, CA, USA) to a 120 µm thickness. The glass plate was immersed in DI water quickly, and the PSU sheet was formed. The as-prepared membrane sheets were washed several times and stored in DI water for 24 h at 4 °C before use.

### 2.4. Preparation of Thin Film Nanocomposite Membrane

The PSU support sheet was first placed on a flat glass plate, on which the residual water was removed by a squeegee roller, and then soaked in the amine solution for 20 s. The aqueous amine solution consisted of 2 wt % MPD, 1wt % CSA/TEA salt, and 0.01 wt % CaCl_2_, all dissolved in Millipore water. The excess amine solution on the membrane was removed using the squeegee roller. The membrane sheet was left to dry for 3.5 min at ambient temperature, and then immersed in 0.14 wt % TMC in an isooctane solution for 15 s to create the polyamide thin film membrane. The sheet was dried in the oven for 6 min at 80 °C and stored in DI water at 4 °C until use.

MCM-41 silica NPs were added to the MPD or TMC solutions, in separate experiments, at different weight ratios varying from 0 to 0.04 wt %. When the NPs were added to the TMC solution, it was necessary to sonicate the mixture for 1 h to achieve full dispersion, while shaking for 15 s was enough when they were added to the MPD solution.

### 2.5. TFN Membrane Characterization

Membrane morphology was examined using SEM (Quanta FEG 600, FEI Company, Hillsboro, OR, USA). The samples were dried at ambient temperature and stored at 4 °C until the test. The membrane samples were coated with platinum using a sputter coater (K575x, Emitech Ltd., Kent, UK) at 20 mA for 1 min to make them conductive for SEM. Different voltage intensities were used depending on the image magnification.

Images of the membrane’s cross-sectional view were taken using a TEM device (JEOL 1400, JEOL Ltd., Peabody, MA, USA). The samples were prepared by immersing the membrane sheets in resin (Eponate 12, Ted Pella, Inc., Redding, CA, USA) and drying. The samples were then cut by Reichert—Jung Ultra cut E ultramicrotome (Reichert, Inc., Depew, NY, USA).

The membrane surface functional groups were detected using attenuated total reflection Fourier transform infrared spectroscopy. A Nicolet 4700 FT-IR (Thermo Electron Corporation, Waltham, MA, USA) system with a multi-reflection smart performer ATR accessory was employed. The collected spectra were from 500 to 4000 cm^−1^ with a 64 scanning rate at a 2.0 cm^−1^ resolution.

The hydrophilicity of the membranes was evaluated by measuring the surface contact angle of DI water. A contact angle video system (VCA-2500 XE, AST products, Billerica, MA, USA) was used for this purpose using a sessile drop method. The measurements were repeated at least six times at different locations of the membrane, and the standard deviation was assessed for the results.

**Figure 1:**
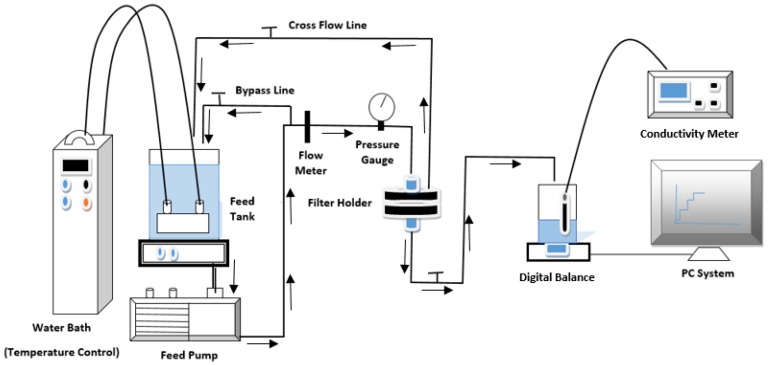
Graphical diagram of the desalination system.

The water flux and salt rejection were measured via a cross-flow filtration system, as illustrated in Figure 1.

The membrane sheet was put in a filter holder (Model: XX4504700, stainless steel, Millipore Corp., Billerica, MA, USA) and tested under 2068 kPa operation pressure for approximately eight hours. The permeate water flux was measured by the weight accumulation in time using LABVIEW software (National Instruments LabVIEW 8.2 with Ohaus digital balance). The water flux was calculated with Equation (1).

(1) $$J=\frac{V}{A\;\times \;t}$$

where *J* is the water flux (L/m^2^·h), *V* the product volume, A the membrane area, and t the accumulation time.

The salt rejection of the brackish water used in this work (2000 ppm NaCl) was estimated by measuring the conductivity of the total dissolved salts using a conductivity meter (HACH Company, Loveland, CO, USA). The measurements were taken at 25 °C, and the rejection was calculated using Equation (2).

(2) $$R\;=\;(1\;-\frac{C_{p}}{C_{f}})\times 100$$

where R is the salt rejection ratio, *C_p_* the permeate conductivity, and *C_f_* the feed conductivity.

## 3. Results and Discussion

### 3.1. Characterizations of MCM-41 NPs

XRD patterns, pore size distribution, the N_2_ adsorption/desorption isotherm, SEM, and TEM have been reported for porous MCM-41 NPs in a previous research in our group [8].

### 3.2. TFN Membrane Characterizations

This study selected 2,2,4-trimethylpentane, also named isooctane, as an organic solvent for TMC. It was used for its intermediate boiling point, low volatility, and lesser health impact, so that the common solvent evaporation problem associated with membrane synthesis could be solved. For comparison, Table 1 presents the properties of isooctane and the traditionally used solvent, hexane [28,29,30,31]. The key is to ensure that the membrane quality could be maintained when hexane is replaced by isooctane.

**Figure 2:**
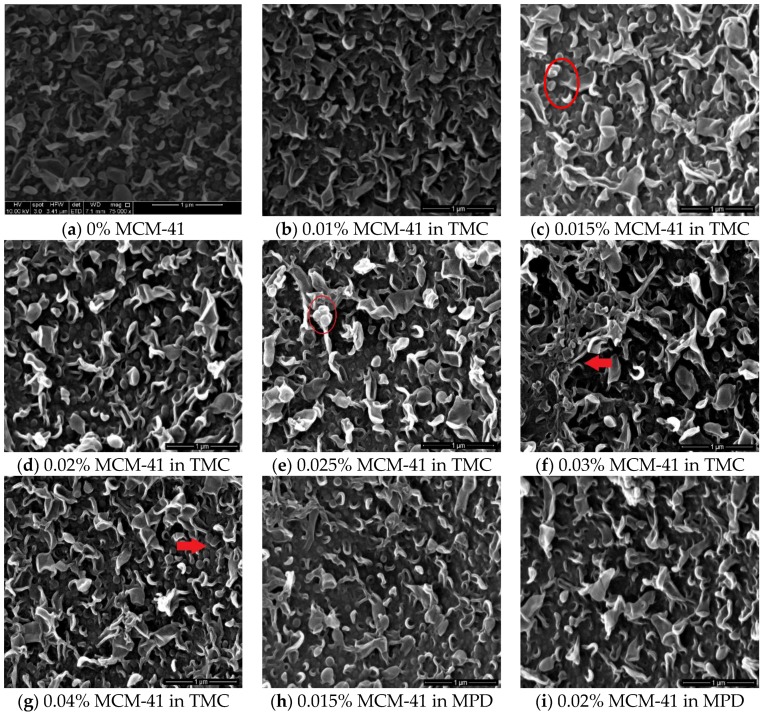
SEM images for membranes with differently loaded NPs: (**a**) TFC; (**b**–**g**) NPs in TMC sol and (**h**–**i**) NPs in MPD sol.

The surface morphology was investigated using SEM. The TFC and TFN membrane morphology with loaded NPs at different percentages are illustrated in Figure 2. It is noted that the control membrane, obtained by the IP reaction between TMC and MPD without additives on a PSU layer, has a leaf-like morphology, Figure 2a, typical of TFC. Adding NPs in different loadings did not change the general structure of the membranes (Figure 2b–i). The added NPs to the TMC solution can be observed as spherical particles within the membrane structure, or by morphology change (Figure 2b–g). By increasing the NP loading, the amount of NPs in the membranes was increased and eventually started to aggregate, as shown in Figure 2g. As is evident from the SEM images, the NPs in the membranes when added via the MPD solution are not as visible as those when the NPs were introduced via the TMC solution. This can be attributed to the fact that the NPs stayed deeper in the membrane since the MPD solution is the first reactant introduced for the IP reaction.

**Figure 3:**
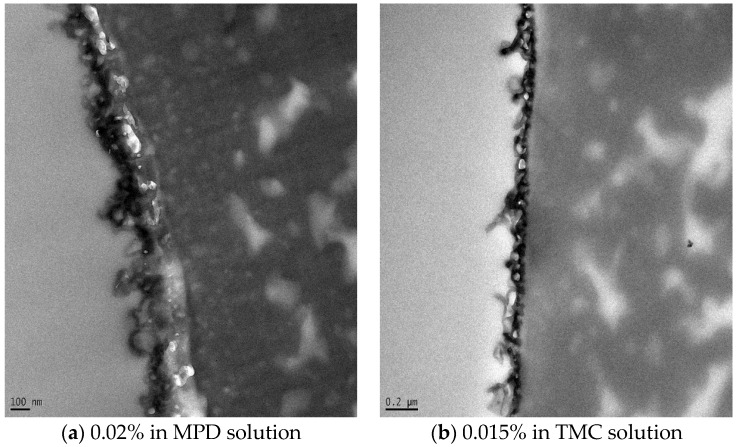
TEM images of the membrane made by adding 0.02% NPs in MPD (**a**) and 0.015% NPs in TMC (**b**).

Figure 3 shows the TEM images of the membrane made by embedding 0.02% silica NPs in MPD solution and 0.015% in TMC solution. The NPs are spherical in shape. It is clear that the particles went deeper inside the membrane layer when they were dispersed in the MPD solution, while they were closer to the surface when dispersed in the TMC solution. This is reasonable since the MPD solution is the first to be poured onto the PSU support layer. Apparently, adding the NPs to the MPD solution created a more stable structure. The thickness of the thin film layer was found to be 100–300 nm, which is somewhat thinner than the membrane previously developed in our group [8]. This difference results from a shorter polymerization time (15 s) used in this study than the previous one (120 s).

**Figure 4:**
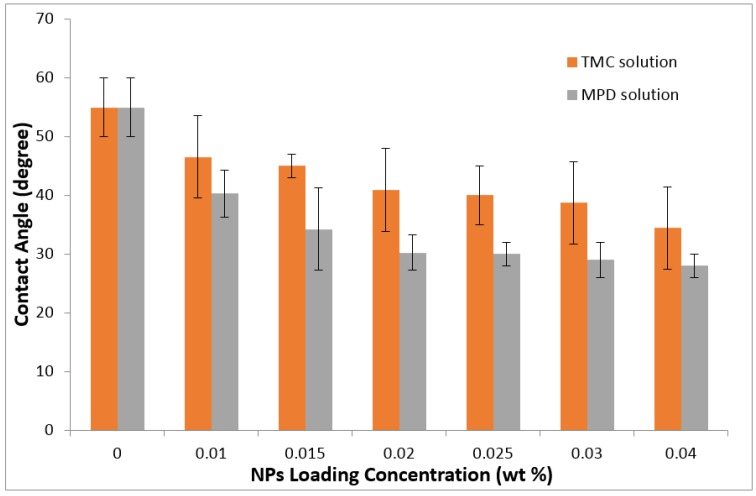
DI water contact angle.

The contact angle measurements are presented in Figure 4. The contact angle was decreased from 54.9° to 34.4° and 28.0° for the membranes embedding silica NPs in the TMC and MPD solutions, respectively. Generally, the membranes made from dispersing the NPs in the MPD solution had lower contact angles than the membranes made from dispersing the NPs in TMC. It is likely that the hydrophilic silica NPs disperse in the aqueous solution of MPD better than in the organic TMC solution, and there is less aggregation when dispersed in the aqueous phase. Dispersing the NPs in an MPD solution would place them deeper in the membrane, so the possibility of NP release would be lower.

**Figure 5:**
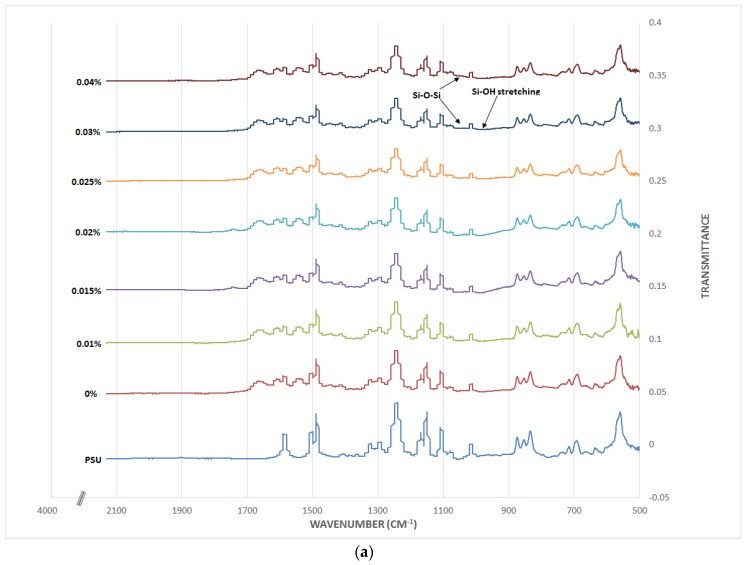

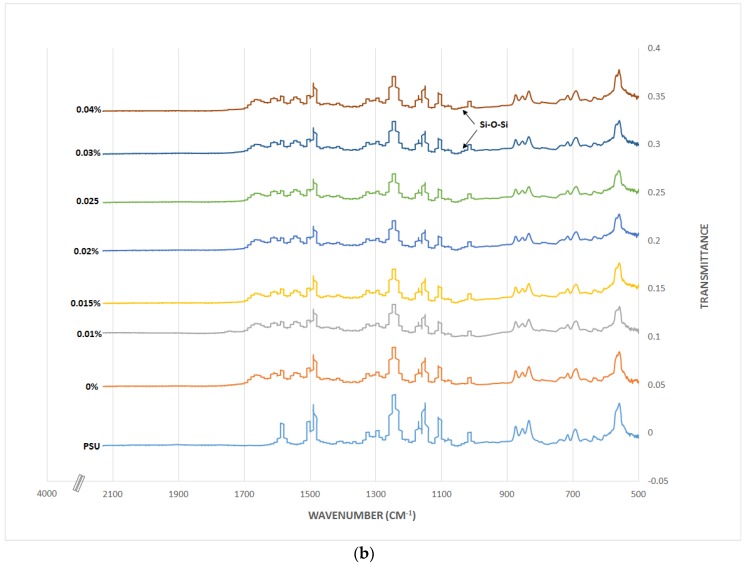
(**a**) Membrane’s ATR FT-IR spectra from embedding NPs in TMC solution; (**b**) Membranes from embedding NPs in MPD solution.

The ATR FT-IR analysis is presented in Figure 5a for the membranes made by dispersing MCM-41 NPs in TMC solution and in Figure 5b for the membranes made by dispersing the NPs in MPD solution. Each also includes the spectra of PSU support layer and original TFC for comparison. Based on the literature, Si–OH stretching vibration is at approximately 950 cm^−1^ [32] and Si–O–Si vibration peak is at approximately 1050 cm^−1^ [24]. When the MCM-41 NPs were embedded at an increasing concentration, there appeared to have tiny peaks corresponding to silica at the highest concentrations, but was still too low to detect with certainty. The other peaks resulted from the PSU layer and TFC membrane chemical bonds. The main vibrations from the PSU were the symmetric and asymmetric O=S=O at 1150 and 1298–1325 cm^−1^, respectively. The carbon bond stretching peaks were at 1245 cm^−1^ for asymmetric C–O–C and 1488–1590 cm^−1^ for aromatic C–C [33,34]. The membrane thin film peaks came from the vibration of C=O stretching (amide І) at 1660 cm^−1^ and C–N stretching and N–H bending (amide П) at 1541 cm^−1^. In addition to that, N–H and C=O stretching peaks were observed at 1610 and 1490 cm^−1^, respectively [8,34,35,36].

### 3.3. Membrane Salt Rejection and Water Flux

**Figure 6:**
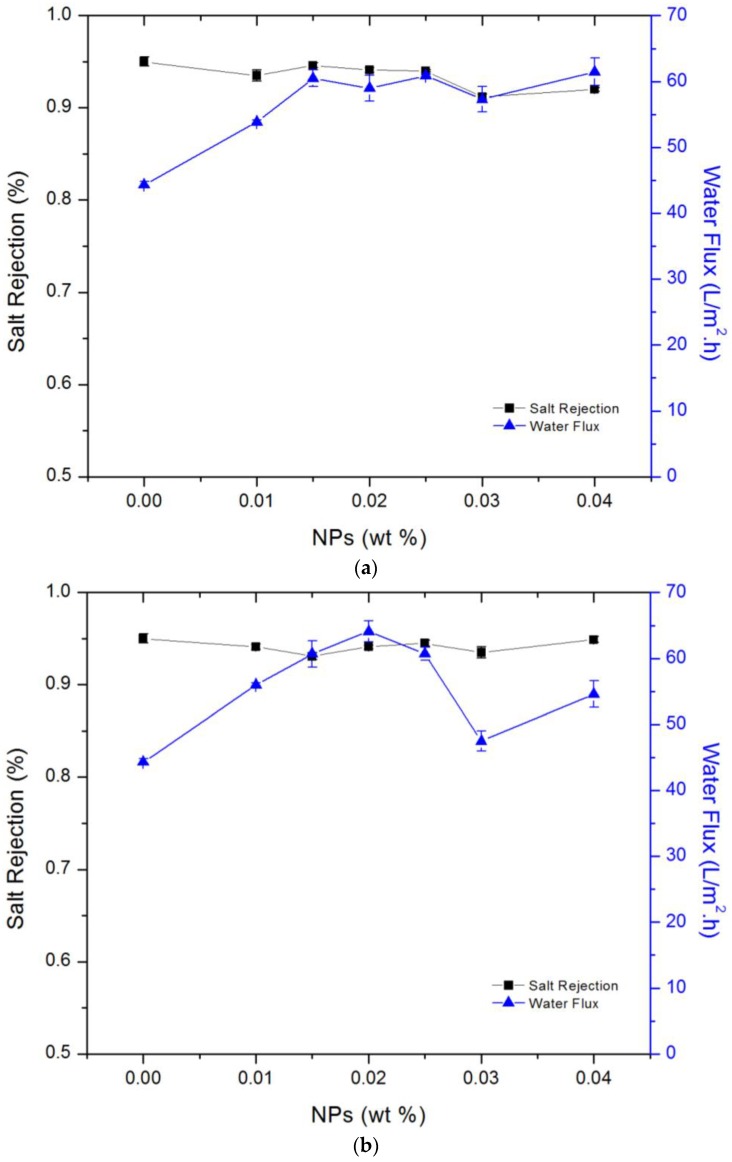
(**a**) Salt rejection and water flux for filling NPs in TMC solution; (**b**) Salt rejection and water flux for adding NPs to MPD solution, under operation conditions of 2068 kPa and 25 °C.

The salt rejection and water flux of the TFC and TFN membranes were studied and presented in Figure 6a for filling MCM-41 NPs in TMC solution and Figure 6b for filling MCM-41 NPs in MPD solution. The system conditions were as follows: a 2000 ppm NaCl solution at 2068 kPa and 25 °C. For the membrane prepared by dispersing MCM-41 NPs in the TMC solution (Figure 6a), the water flux increased as the NP percentage increased, while the salt rejection slightly decreased. The concentration range of NPs was 0%–0.04% for both solutions. The optimal loading of the silica NPs to TMC solution was 0.015%, a condition under which the water flux was increased from 44.33 to 60.5 L/m^2^·h compared with the TFC membrane, while the rejection remained essentially the same (from 95.0% to 94.6%). Adding the NPs to the MPD solution had a comparable effect; under an optimal NP concentration of 0.02%, the water flux increased from 44.33 to 64.1 L/m^2^·h in comparison with the TFC membrane, while the rejection was decreased from 95% to 94.1% (Figure 6b). Dispersion of NPs in the MPD solution yielded a slightly better and more stable result compared to the addition of NPs to the TMC solution. The effect of the NPs likely comes from the hydrophilic nature of the mesoporous silica, and a more hydrophilic surface may cause an increase in the water flux [8]. A slightly higher water flux associated with the addition of silica NPs to the MPD solution compared to the TMC organic solution is consistent with the contact angle measurements shown in Figure 3. Dispersing NPs in the MPD solution could also help place the NPs deeper into the membrane, resulting in a more stable structure.

To place our research in the context of other reported nanocomposite membranes, we tabulated the optimal solid loading, water permeability, and NaCl rejection in Table 2 for a number of nanocomposite membranes. The comparison indicated that the MCM-41 silica nanocomposite membranes had a rejection comparable to others but a significantly higher water flux.

## 4. Conclusions

Isooctane was used in this study as a TMC solvent due to its higher boiling point and lower volatility than hexane for thin-film composite preparation, showing no effect on membrane performance. A comparison was made between dispersions of MCM-41 silica NPs in MPD and TMC solutions. The results show that the performance of membranes made by dispersing the silica NPs in the MPD solution was slightly better than those made by dispersing the NPs in the TMC solution. At an optimal dosage of 0.02% MCM-41 NPs in the MPD solution, the water flux increased from 44.3 to 64.1 L/m^2^·h, while the salt rejection decreased slightly from 95.0% to 94.1%. At an optimal dosage of 0.015% MCM-41 NPs in the TMC solution, the water flux increased from 44.3 to 60.5 L/m^2^·h and the salt rejection remained essentially the same.

## Figures and Tables

**Table 1:** A comparison of physical properties between isooctane and hexane.

Property	Isooctane	Hexane
Boiling Point (°C)	99	69
Flash Point (°C)	−12	−26
Evaporation Rate	1	15.8
Vapor Pressure (mmHg)	88 (at 37.80 °C)	256 (at 37.70 °C)
Vapor Density (Air = 1)	3.94	2.97
Relative Density (g/cm^3^)	0.690	0.659
Health Hazard	Less	Higher

**Table 2:** A performance comparison of thin film nanocomposite (TFN) membranes.

Nanofiller	Optimum Loading (wt %)	Water Permeability (L/m^2^·h·kPa)	NaCl Rejection %	Reference
MCM-41	0.02	0.0310	94.1 ± 0.2	This work
Graphene oxide	0.015	0.0287	93.8 ± 0.6	[37]
Multi wall nanotubes	0.1	0.0175	~90	[38]
Zeolite	0.4	0.0137	93.9 ± 0.3	[39]
Silica	0.6	0.0087	95.1	[25]
